# Design and Implementation of an Integrated Competency-Focused Pharmacy Programme: A Case Report

**DOI:** 10.3390/pharmacy7030121

**Published:** 2019-08-27

**Authors:** Theo J. Ryan, Tamasine Grimes, Martin C. Henman, Eimear Ní Sheachnasaigh, Máire O’Dwyer, Cicely Roche, Sheila A. Ryder, Astrid Sasse, John J. Walsh, Deirdre M. D’Arcy

**Affiliations:** School of Pharmacy and Pharmaceutical Sciences, Panoz Building, Trinity College Dublin, The University of Dublin, Dublin 2, Ireland

**Keywords:** competence, curriculum development, curricular integration, experiential learning, pharmacy education

## Abstract

This paper describes the design and implementation of elements of an integrated competency-focused pharmacy programme in the School of Pharmacy and Pharmaceutical Sciences (SoPPS), Trinity College Dublin (TCD), Ireland. Following a national review of pharmacy education and training in Ireland in 2010, and subsequent publication of legislation in 2014, the School has implemented a five-year integrated programme of pharmacy education and training, leading to the award of a Master’s degree in Pharmacy (M. Pharm.). Curricular integration has been achieved by underpinning the new programme with a national competency framework for pharmacists and through the utilisation of curricular integration themes. Programme integration also encompasses embedded experiential learning placements in Years 2, 4 and 5 of the five-year programme. The new five-year integrated pharmacy programme, which commenced in 2015, replaced the 4 + 1 model of education and training where a four-year Bachelor’s degree was followed by a one-year internship, which was a distinct and separate element of the students’ training.

## 1. Introduction

The regulator of the pharmacy profession in Ireland, and the accrediting body for pharmacy education and training, is the Pharmaceutical Society of Ireland (PSI). A PSI-commissioned review of pharmacy education, training and accreditation models in Ireland, the Pharmacy Education and Accreditation Reviews (PEARs) project report, was published in 2010 [1]. It recommended replacing the four-year Bachelor’s degree plus the one-year internship leading to M. Pharm. qualification (4 + 1 model), where the internship was the regulator’s responsibility, with a five-year programme with the internship integrated as a series of placements. The PSI consequently required an integrated five-year programme leading to the award of a Master’s degree as the qualification required for application for entry to the register. National legislation and accreditation standards from the regulator enacted these changes and transferred responsibility to all three Schools of Pharmacy in Ireland (School of Pharmacy and Pharmaceutical Sciences (SoPPS), Trinity College Dublin (TCD); School of Pharmacy, Royal College of Surgeons in Ireland (RCSI); School of Pharmacy, University College Cork (UCC)) for assuring the suitability of their graduates to apply to enter the register [2,3,4]. Based on the PEARs report, the PSI in collaboration with the relevant national pharmacy stakeholders, known collectively as the National Forum for Pharmacy Education and Accreditation, oversaw the recommended design changes to education and training of pharmacy students in Ireland. Membership of the National Forum consisted of representatives from the three Schools of Pharmacy; pharmacists from the main practice settings of community, hospital and industry; patient and student advocates; the PSI; healthcare related government representatives; and an external international expert. 

In order to meet legislative requirements, standards and the vision and academic values of the SoPPS, TCD, the content, delivery and sequencing of the curriculum to align teaching and learning with student and pedagogic needs in the new programme were revised, a process informed by further specific consultation with the School’s graduates and members of the profession. Given their central relevance to the new five-year integrated programme curriculum, three specific elements have been identified as the focus of this paper: The competency framework relevant to the practice of pharmacy in Ireland, curricular integration and experiential learning placements. This paper describes the design and implementation of these elements of a new competency-focused five-year integrated pharmacy programme in the SoPPS, TCD, which had its first student intake in 2015.

## 2. Design

### 2.1. Competency Framework

A recommendation from the PEARs project was to underpin the new integrated programme by education and training standards based on the competence required at the point of professional registration. Subsequently the PSI published their Core Competency Framework for Pharmacists (CCF) which sets out the competencies, including knowledge, skills, attitudes and behaviours required of pharmacists in their everyday practice and therefore also to be attained by students pursuing pharmacy education and training in Ireland [5]. The CCF is based on a global competency framework drafted by the Pharmacy Education Taskforce, which is a collaboration of the International Pharmaceutical Federation (FIP) with the World Health Organization (WHO) and United Nations Educational, Scientific and Cultural Organization (UNESCO) [6]. The CCF is divided into six overarching domains of practice: *Personal Skills*; *Professional Practice*; *Safe Supply of Medicines*; *Safe and Rational Use of Medicines*; *Public Health* and *Organisation and Management Skills*, and comprises 25 competencies that are expected of a pharmacist. Each competency is further demonstrated by specific behaviours. 

In the programme described here, competence development in the context of the CCF includes a purpose-designed approach termed CCF Live that focuses on the recognition and demonstration of competencies in simulated and actual practice environments. CCF Live was designed as a reflective exercise, which requires students in Years 1–3 to observe competency demonstration in practitioners and to consider their own development and learning needs against the CCF, having regard to both formal and informal practical activities. CCF Live ensures familiarisation and engagement with the CCF which constructively aligns with the summative competency assessment (CA) in Years 4 and 5, where the students are required to demonstrate consistent performance at level 3 (mostly) and level 4 (always), respectively, across all relevant domains of the CCF. These CCF activities are underpinned by adult learning theory, whereby the student is enabled to move from a relatively dependent pedagogical status to the self-directed andragogical position that is desirable for an adult learner [7]. 

### 2.2. Integrated Curriculum

Reference to curricular integration relevant to pharmacy education has been notable in the literature over the past decade [8,9,10,11,12,13,14,15,16,17,18,19,20]. In common with accreditation standards in other jurisdictions, the PSI’s accreditation standards for the five-year integrated pharmacy programme include reference to curricular integration, such as in Standard 4: “The curriculum must be planned to deliver an integrated learning experience that combines and co-ordinates all components in a logical and cohesive manner…” The provider must also show how the curriculum “is progressive in dealing with issues in increasingly complex and interrelated ways so that graduates meet the Core Competency Framework for Pharmacists” [21]. High-level policy development and oversight is facilitated through the School’s Strategic Advisory Board which consists of practitioners from all areas of the pharmacy profession (hospital practice, community practice, industrial and regulatory).

An outline of curriculum content comprising the modules in each year of the programme is provided in Appendix A. The programme is designed to foster integrative learning through a series of modules across the five years that are integrated both horizontally and vertically. A number of desirable characteristics of the approach to integration were recognised, as follows: (a) that the integration approach would be programme-focused, to allow for both intra- and inter-module integrative learning across the whole programme, accounting for different levels of integration within modules; (b) that the approach to integration would align with both the CCF and the educational ethos of the SoPPS in TCD; (c) in being cognisant of the resource implications of designing and implementing a new curriculum, that the curricular integration approach would remain applicable through the evolution of the curriculum and inevitable associated curricular changes; (d) that the approach to curricular integration would support the development of visual aids to facilitate integrative teaching and learning for both students and staff. Therefore, to enable a systematic and sustainable approach to integrative teaching and learning in the curriculum, an integration co-ordinator role was established. The integration co-ordinator established and led an integration working group and a visual and curricular integration themes group, to establish the SoPPS’ approach to integration. The aim of the integration approach was twofold:

(1) To facilitate both the depth of disciplinary learning required to understand and develop concepts of pharmacy science and practice, and cross-disciplinary integrated and integrative teaching to relate learning content to practice experiences and settings. This was addressed by including modules of differing levels of integration, as appropriate to the module, throughout the programme.
(2) To allow flexibility in the curriculum design to enable it to evolve with the new programme, and to accommodate resource challenges such as timetabling constraints. This was addressed by using a thematic approach to sequencing, whereby core curricular themes were identified and modules were aligned with one or more themes, along with methods for comprehensive yet flexible cross-referencing of relevant material between modules. Evolution of curricular design and structure can be facilitated through module-theme realignment as necessary. The thematic approach also facilitates mapping of the curriculum to the CCF.

Harden’s integration ladder is utilised in module descriptors to illustrate the level of integration for each module of the programme, with lower levels corresponding to more specific discipline-based teaching and higher levels corresponding to teaching and learning, which is increasingly cross-disciplinary and applied [22]. Each module is also mapped to the relevant competencies of the CCF.

**Figure 1:**
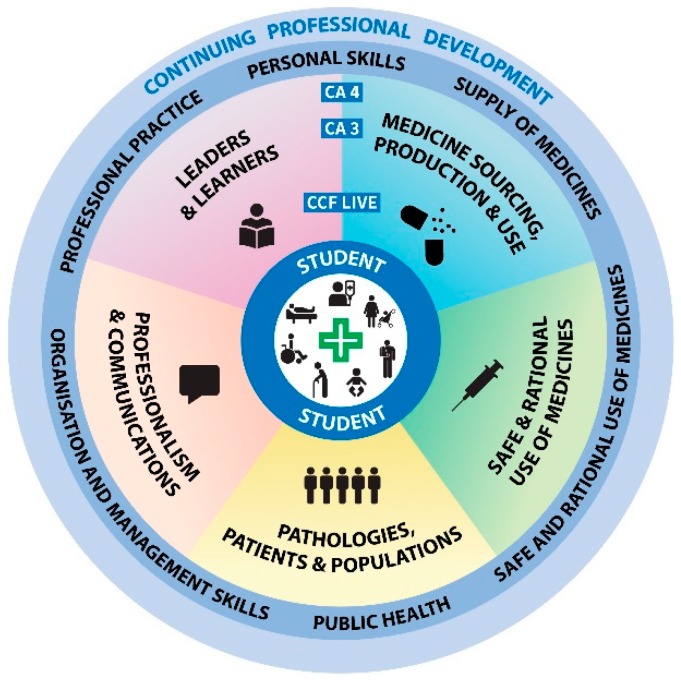
Representation of the curriculum of the five-year integrated pharmacy programme showing the School’s five curricular integration themes underpinned by the six domains of the PSI’s Core Competency Framework for Pharmacists. This visual also highlights the role of competency assessment and reflective learning to support post-qualification continuing professional development as central elements in the curriculum. CCF: Core Competency Framework for Pharmacists. CA3: Competency Assessment at level 3 (‘mostly’). CA4: Competency Assessment at level 4 (‘always’).

The thematic approach to curricular integration and sequencing was designed to consider the breadth of disciplines in pharmaceutical science and practice contributing to the curriculum, the professionalization of the student and the research-led ethos of the school. The following five core integration themes were identified: 1. *Medicines Sourcing, Production and Use*; 2. *Safe and Rational Use of Medicines*; 3. *Pathologies, Patients and Populations*; 4. *Professionalism and Communications*; 5. *Leaders and Learners.* All modules in the programme are mapped to relevant curricular integration themes. Furthermore, the themes are also aligned with competencies according to the CCF. The curricular themes, their definition and scope and associated visual representation of the curriculum (Figure 1) were generated by the visual and curricular integration themes group of academics in the SoPPS.

As shown in Figure 1, the curriculum is patient-centred and the student’s developmental journey involves the five integration themes. The six domains of the CCF surround the curriculum illustrating the development of a competent practitioner. The importance of post-qualification life-long learning is reflected in the curriculum through continuing professional development (CPD), for which students are prepared through development of their reflective practice.

### 2.3. Experiential Learning

Within the 4 + 1 model of pharmacy education and training, although the school had incorporated work experience into the programme the only legally required period of time in practice placement for students had been the one-year internship in which students undertook a structured Masters in Pharmacy (M. Pharm.) following the four-year Bachelor’s degree. The one-year internship was a standalone national programme for which the PSI as the regulator had overall responsibility, with one School of Pharmacy contracted and accredited to deliver the programme to graduates from any of the three Schools of Pharmacy in Ireland. 

For the five-year integrated programme, the PEARs recommendation for the integration of experiential learning placements was structured by the National Forum as three periods. TCD students spend two weeks on experiential learning placement in Year 2, followed by a four-month placement in Year 4 and an eight-month placement in Year 5. Placements in Year 2 are undertaken in community and hospital practice settings. Year 4 placements are undertaken across a breadth of practice settings including industry, regulatory, hospital and community; a patient-facing environment is not required in this year. In contrast, Year 5 placements are legally required to be undertaken in a patient-facing practice setting, namely community or hospital practice. The dispersal of experiential learning placements across the five years of the programme is intended to ensure contextualization of student learning at an early stage of their education and training journey, facilitate integration and reflection in the curriculum following the Year 2 and Year 4 placements, and offer students an opportunity to experience a variety of practice settings, where possible.

### 2.4. Assessment

The semesterised academic year structure in the SoPPS facilitates a wide range of assessment modalities, including standard end of semester assessments in addition to in-term continuous assessment components. Assessment is conducted at module level, aligned to module learning outcomes, with module outcomes then mapped to programme outcomes. Assessments *for* and *as* learning have been included within the five-year integrated programme to engender student learning or to enable students to become more aware of how they study, in addition to assessments *of* learning primarily intended to measure the achievement or degree of student learning. The forms of assessment are commonly refined in response to curriculum changes, developments in accreditation standards, scientific/technical/scholarly progress, external examiner or student feedback, professional matters and changes to pharmacy practice. Assessment is aligned with the programme’s teaching and learning strategy and assessment strategy. A key element of the assessment process in the new programme is formal evaluation of students’ attainment of competencies in the practice settings, culminating in consistent demonstration of all behaviours within the six domains of the CCF.

While integrated assessments must sit within a module due to the university’s modular programme structure, the specified learning outcome under assessment can require drawing on learning from other modules, thus facilitating integrated assessment. Examples of integrated assessment include simulated/real patient care within dispensing classes, Objective Structured Clinical Examinations (OSCEs), case- and poster-presentations and the competency assessments administered by the placement preceptors. 

Comprehensive competence demonstration across three domains at CA3 level in Year 4 is a requirement for progression to Year 5, whereas previously only a limited (and variable) set of competencies were assessed in campus-based assessments such as practical tests and OSCEs. Hence there is greater assurance of competency at that stage of the new programme compared to Year 4 of the old programme, although the threshold to be met at the end of Year 5 (CA4 across all competencies) is the same for both programmes.

## 3. Implementation

### 3.1. Competency Framework

Commencing in Year 1 of the programme, students become familiar with the CCF through its introduction in the first pharmacy practice module and participation in a CCF workshop. The teaching and assessment process enables students to engage with competency-based learning in a progressive and iterative manner, through all learning experiences. Through CCF Live, students are required to undertake reflective practice with documentation in a personal e-portfolio of how various pharmacy practices that they observe and/or practise in the course of the programme (including professional, clinical and more basic pharmaceutical science content) map to the domains, competencies and behaviours of the CCF. Student reflective practice is encouraged through completion of CPD cycles in a self-directed manner, with specific prompts after activities such as external site visits, structured experiential learning placements, and OSCEs as they progress through the programme. The process of undertaking reflective practice helps students to appreciate the competencies required of the pharmacist. Furthermore, the CPD cycles within the programme train students in self-appraisal, the formulation of plans to address their learning needs, documentation of their progress and recognition of its impact on practice in preparation for post-registration CPD requirements [2].

### 3.2. Integrated Curriculum

By assigning relevant curricular integration themes to modules, the curricular sequencing from more basic to more applied content and its associated assessment is evident. As the school’s integration themes are specifically designed to facilitate curriculum module sequencing and integration, there is no separate assessment of the curricular themes. Content relevant to the curricular themes is assessed at module level, with module learning outcomes mapped to programme learning outcomes, enabling an overview of programme-level assessment. Furthermore, as each theme is aligned to the CCF domains, competency assessment is also relevant to the curricular themes. 

The themes support both the development of the student as an integrative learner, and academic staff in inter-module integration through appropriate cross-referencing. Furthermore, the infographics associated with each relevant theme (Figure 1) are presented with each module descriptor, facilitating easy identification of modules with similar themes in the programme guidance documentation. For example, in the third year of the programme the module entitled Respiratory and Gastrointestinal Systems and Clinical Therapeutics addresses three of the integration themes, namely *Medicine Sourcing, Production and Use*; *Pathologies, Patients and Populations* and *Safe and Rational Use of Medicines*, thereby enabling horizontal and vertical integration with similarly themed modules across the programme. In contrast, the practice of pharmacy modules across all five years of the programme map to all five integration themes enabling students to contextualise their learning in all modules with that of professional practice.

In terms of module integration levels based on Harden’s integration ladder [22], an example of a highly integrated module (i.e., levels 9–11) is the third-year module Respiratory and Gastrointestinal Systems and Clinical Therapeutics. In this module, a number of topics from traditional disciplines, including pharmacology, pharmacognosy, pharmaceutical chemistry, pharmaceutics and practice of pharmacy are taught in an integrative manner in one module, and student assessment includes integration of material through poster presentations and case-based clinical pharmacy assessment. The assigning of integration levels enables identification of modules where deeper discipline-specific content is delivered and assessed, and modules where integrative application of knowledge is expected and assessed, representing lower and higher integration levels respectively. 

**Figure 2:**
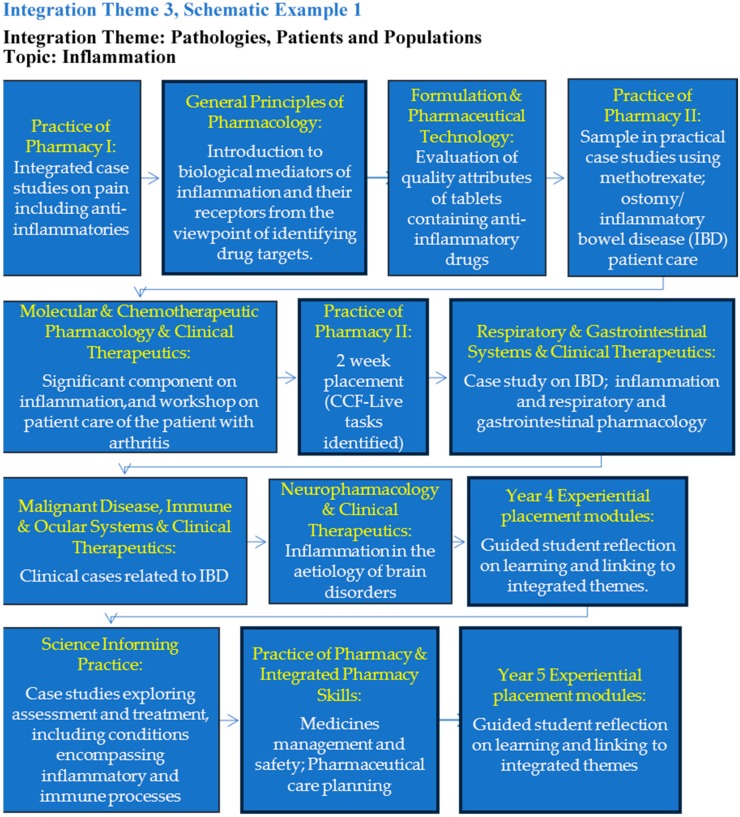
Sample integration schematic visual tool on the topic of *inflammation* within the curricular integration theme of *Pathologies, Patients and Populations* and the associated horizontal and vertical integration across the programme. CCF: Core Competency Framework for Pharmacists.

To further facilitate thematic curriculum sequencing and integrative learning, students are provided with sample topics as visual tools within each curricular integration theme. An example is the topic *inflammation* within the curricular theme *Pathologies, Patients and Populations* (Figure 2). The sample topics are presented as schematic diagrams, which highlight relevant content in associated modules, including activities in experiential placement modules. These topics aim to demonstrate to students how teaching and learning content is horizontally and vertically integrated within a year and between the years respectively.

To underpin the programme-focused approach to integrative learning, upon introduction of each module and in the module descriptors, module information is systematically provided to the students concerning (a) the curricular themes associated with the module; (b) the relevance of a module to sample topics within each theme (and thus other modules associated with that topic) and (c) general topics of cross-relevance to other modules (both within the same year and in other years of the programme).

### 3.3. Experiential Learning

To enable the successful implementation of the dispersed experiential learning placements in the five-year integrated programme, two major changes to resources were required. The first of these was the establishment of a shared service across the three Schools of Pharmacy in Ireland for the sourcing, management and quality assurance of experiential learning placements through what is known as the Affiliation for Pharmacy Practice Experiential Learning (APPEL) [23]. Responsibilities of APPEL include the centralised matching and allocation of placements to students, the development of training and support materials for trainers and students while on placement, the operational management of experiential learning placements and post-placement survey and evaluation. Through the work of APPEL, the sourcing, allocation and quality assurance of the experiential learning placements is nationally centralised.

The second change has been the introduction of APPEL Practice Educators whose responsibility is to bridge the gap between the Schools of Pharmacy and experiential learning training establishments by supporting both the students and the preceptors throughout the experiential learning journey. Students are prepared in advance of their placements by the Practice Educators, who guide the students through the set placement activities and expectations of professional behaviours. Provision of support to students and preceptors while on placement is through site-visits as well as through telephone or email contact.

The two-week Year 2 experiential learning placement is undertaken in a community or hospital practice setting, which allows students to gain an early patient-facing experience while under close supervision by a preceptor. The Year 2 placement is primarily observational in nature due to the early stage of student education and training. Learning and assessment are enabled through placement specific activities, which incorporate calculations, competency familiarisation, communication skills and reflective practice. Students document these activities in an online portfolio, which contributes to their experiential learning assessment. Following placement, a class debrief conducted by the Practice Educator provides students with the opportunity to share their collective experiences, thereby prompting further reflection.

The four-month Year 4 experiential learning placement can be undertaken in any pharmacy-related placement setting with approved supervision, including community practice, hospital, industry, academia, regulatory bodies and other areas where roles are emerging for pharmacists (e.g., within a pharmacoeconomic centre). While on the Year 4 placement, students are required to undertake three online modules tailored for experiential learning, which are aligned to three of the six CCF domains, namely *Personal Skills*; *Professional Practice*; and *Organisation and Management Skills* [24]. As the process for pharmacy experiential learning across the three Schools of Pharmacy in Ireland is nationally co-ordinated through the shared service of APPEL, the online placement modules are co-developed and co-delivered by the three Schools. Assessment of the online placement modules is through a combination of academic assessment (students’ individual and group work activities) and competency assessment by preceptors. Preceptor pharmacists are integral to supporting students’ development within the experiential learning placements and in assessing their performance against specific competencies of the CCF. In Year 4, students are assessed for competency by their preceptors across the same three CCF domains as the online placement modules (*Personal Skills*; *Professional Practice*; and *Organisation and Management Skills*) and are required to achieve a competency level 3 out of 4 for each relevant behaviour (CA level 3).

The first wave of Year 5 experiential learning placements will commence in January 2020. The eight-month Year 5 placement is undertaken in a community pharmacy or a hospital pharmacy practice setting only as it must be patient-facing. Students are required to undertake three online modules while on placement, on this occasion with all six domains and associated competencies of the CCF being addressed. As with the Year 4 placement, academic assessment of the online modules is through individual and group activities. Regarding competency assessment, students will be assessed by their placement preceptor across all six CCF domains and are required to achieve a level 4 out of 4 for each competency (CA level 4), thereby demonstrating competence prior to the professional registration examination leading to first registration as a pharmacist.

### 3.4. Quality Assurance–General

There are four main strands of quality assurance that are relevant to the School and to the integrated pharmacy programme:

- TCD is a member of the Irish Higher Education Quality Network and is externally assessed by Quality and Qualifications Ireland, the state agency responsible for promoting quality in the education sector.
- Since the integrated pharmacy programme leads to a professional qualification, it is subject to the standards of accreditation set out by the regulator, which must be satisfied by the School and the University [3].
- Within the University a seven-year cycle of quality review is in place for all Schools, guided by national legislation, and this assures the academic standards and operation of the School as a whole [25,26]. The University, through its Quality Office and the Faculty of Health Sciences, continuously monitors the quality of the programme delivered by the School using information derived from key performance indicators and student feedback.
- The School’s quality management system is supported by the School’s Programme Management Committee, which has responsibility for monitoring, reviewing and making recommendations on the design and implementation of the curriculum. Through this committee the School uses standard quality assurance methods to guide and implement programme changes to teaching and assessment including external examiner’s reports, external expert steering group reports, student and staff feedback, and annual review of all modules.

### 3.5. Quality Assurance of Experiential Learning

As the experiential learning placements are embedded and integrated throughout the five-year programme, the responsibility for assuring and enhancing the quality of the experiential learning placements lies with the individual Schools of Pharmacy and APPEL, rather than with the PSI as regulator as had been the case with the previous 4 + 1 programme. APPEL undertakes this quality assurance role on behalf of the three Schools of Pharmacy in Ireland and has implemented a quality assurance process for all placements. Students, preceptors and the associated training establishments are all required to engage in the governance processes.

Quality assurance mechanisms to support effective governance of the placement programme include the implementation of policies relating to accreditation of preceptors and the training establishments, accreditation appeals processes, placement visits, and documentation and review of patient safety incidents and breaches of the Student Code of Conduct. Placement agreements between APPEL and the training establishments detail placement requirements including insurance, induction, health and safety requirements, the suitability of the placement environment and that the training establishment has the capacity to provide student(s) with adequate supervision. Through its accreditation process, APPEL can be assured that preceptors and training establishments engaged in the programme meet statutory requirements pertaining to experiential learning placements and other relevant legislation [27,28].

As part of its commitment to quality, APPEL prepares both students and preceptors for the placement experience. Practice Educators act as the interface between the shared service of APPEL, the three Schools of Pharmacy and the training establishments. One of the main roles of a Practice Educator is to prepare preceptors and students in advance of the placements. This is achieved through delivery of both face-to-face and online training for preceptors and via dedicated placement-specific lectures, workshops and preparation days for students. Regarding evaluation and quality improvement, stakeholder engagement is ongoing, and feedback is encouraged both in a structured manner (e.g., via Practice Educator placement visits, post-placement surveys and content review) and on an ad hoc basis via communication with the Practice Educators and the broader APPEL team. In addition, external benchmarking of student performance is enabled by the co-delivery of the online placement modules with the other two Schools of Pharmacy for Years 4 and 5 of the programme.

## 4. Discussion

Mapping of all modules to competencies of the CCF has enabled systematic demonstration and evaluation of all competencies to be addressed at module level and facilitates gap analysis and content changes where necessary. The CCF can therefore be utilised in a formative manner to foster familiarity in students with the required competencies for practice and to develop their competence while simultaneously preparing them for post-qualification requirements [2,29].

Curriculum sequencing has been identified by O’Neill et al. as a curriculum component not optimally explored in higher education, and it has been suggested that sequencing can be arranged using numerous frameworks including linear, spiral, thematic and student-centred approaches [30]. While it has been proposed that modularisation, among other factors, can lead to the fragmentation of curricula [31], it can be considered that appropriate curricular sequencing supports curricular integration. While having a collective philosophy amongst academic staff is important for the development of a sequenced programme, on its own it is not sufficient [30]. In the SoPPS, TCD, utilisation of curricular integration themes across the programme, has greatly assisted in mapping the curriculum and ensuring coherent sequencing to students and staff members alike. The use of curricular integration themes aligned to a competency framework also helps to provide a solid structure to the overall programme, as identified by Neary [32]. Whereas revision of modern pharmacy curricula will often include an emphasis on curricular integration, there exist numerous approaches to the degree of integration and integration types and methods used as identified in a recent comprehensive scoping review on curriculum integration in health profession programmes by Kerr et al. [20]. However, this review has also revealed that there are few literature examples focusing on a whole-programme thematic sequencing approach to integration in pharmacy curricula, in particular where themes are not confined to a body-systems/disease state approach, but rather using multiple cross-cutting themes and topics (which can include body systems/disease states). Furthermore, the approach presented in the current work aligns curricular integration themes with the CCF, and implicitly links didactic teaching, experiential learning and post-qualification practice (to which the CCF applies).

It has been suggested that the development of strong building blocks, such as core professional modules, within a curriculum gives greater structure to its overall sequencing. Furthermore, developing such building blocks is enhanced by external drivers such as professional accreditation and competency frameworks [30]. Consequently, it is anticipated that the structure of the five-year integrated programme will benefit from ongoing professional accreditation and use of the CCF as a basis for integration.

One intention behind developing and implementing the SoPPS’ curricular integration themes is to enable the course curriculum to go beyond the competencies required by the CCF; thus the curricular themes can be considered to be broader in scope than the domains of the CCF. As an example, the curricular theme of *Leaders and Learners*, while addressing CCF domains such as *Professional Practice* and *Personal Skills*, will also enable student preparation for the ever-evolving future roles of the pharmacist. Furthermore, reflecting the research-led teaching ethos of the SoPPS, the *Leaders and Learners* theme encompasses the global perspective of pharmacy prevalent within the School. For example, various modules include emphasis on global health and regulation across multiple jurisdictions, thus underpinning a broader interpretation of pharmacy practice in the curriculum than that applicable in the CCF.

The integrated programme is designed to facilitate high quality experiential learning throughout, commencing at an early stage, with a view to achieving two main outcomes. The first is to enable students to contextualise their theoretical and academic learning and thereby understand how to apply knowledge and skills in practice. The second is to develop student competence prior to professional registration. Furthermore, students will be able to experience multiple sectors of practice over the course of their experiential learning placements. Through the SoPPS’ programme integration co-ordinator, the experiential learning placement modules’ design and activities are shared among academic staff involved only in campus-based curriculum delivery. This helps to facilitate identification of all relevant material to placement activities, and underpins inter-module integration of campus-based and experiential learning placement modules, through prospective and retrospective cross-referencing. All experiential periods therefore build on a theoretical framework and knowledge base developed throughout the non-experiential (campus-based) periods of the programme. Placement-based experiential learning is fundamental to supporting the student to achieve the programme learning outcomes and to proceed to undertaking the terminal professional registration examination to facilitate entry to the register.

### Challenges

The design and implementation of the five-year integrated pharmacy programme curriculum represents a significant development in pharmacy education in Ireland. Notable advantages of the new programme in the SoPPS include the facilitation of quality assurance of experiential placements through APPEL, the framework for curricular integration with a flexible design to evolve with the programme, and the systematic embedding of the CCF within the curriculum. However, as expected with the implementation of a novel curriculum of this magnitude, challenges have been identified. As highlighted by other studies, implementation of an integrated curriculum requires considerable time and resources [8,20].

At a national level, the establishment of the National Forum for Pharmacy Education and Accreditation, involving stakeholders from all sectors of the profession (community, hospital, industrial and regulatory roles), as well as patient and student representatives, helped to facilitate a coherent approach to the development of a legislatively required integrated programme of pharmacy education and training.

At a school level and from the perspective of curriculum design, challenges include identifying the optimal balance between time dedicated to deeper disciplinary learning and time allowed for integrative learning [8]. Logistics, workload, faculty buy-in and concern around the marginalisation of foundation sciences are among the main challenges to curricular integration reported by Poirier et al. in a survey on approaches and attitudes to curricular education in pharmacy schools in the United States [10]. The establishment of the integration working group and visual and curricular themes working group in the SoPPS enabled some of the logistical challenges to be addressed, and resulted in the development of suitable cross-cutting curricular themes as a valuable resource to underpin curriculum integration. It should be noted, however, that additional value arose from the cross-disciplinary nature of these working groups, in the facilitation of discussion around increased inter-disciplinary design and integration of teaching content. Furthermore, the viability of the whole-programme integration approach is evidenced by the fact that following the inevitable curriculum changes that occur on implementation of a new programme, updating of module-theme alignment and sample schematics (Figure 2) as part of annual module review are sufficient to render the curriculum integration continuously up to date, and are managed by the integration co-ordinator. As we enter Year 5 of the new programme, no fundamental change to the curriculum integration visuals, themes or approach has been required. Thus, presenting this sustainable, flexible approach to thematic integration, which recognises the necessary breadth and depth of disciplines relevant to pharmacy education, along with the significant time constraints which many academic faculty staff work within while undertaking curriculum revision, should support efforts by other programme providers on curriculum revision.

Regarding the experiential learning components of the programme, moving from a 4 + 1 model of education and training to a five-year integrated programme with embedded experiential learning placements created a greater need for placement numbers in a given academic year, rising from approximately 180 per year across all three Schools of Pharmacy nationally (one-year internship), to almost 600 placements (Years 2, 4 and 5). A consortium agreement between the three Schools of Pharmacy in Ireland leading to the formation of APPEL as a shared service for the sourcing, management and quality assurance of experiential learning placements is crucial to the success of the programme to date. Central to the success of APPEL is the role of the Practice Educators in sourcing sufficient quality assured placements and ensuring adequate training and supports for students and preceptors.

## 5. Conclusions

An integrated programme of pharmacy education and training has been developed using curricular integration themes and embedded experiential learning within practice placements, underpinned by a national competency framework for pharmacists and supported by robust quality assurance activities. The flexible, cross-cutting thematic approach to curricular integration implemented by the SoPPS, TCD facilitates a sustainable approach to curricular integration and inclusion of curricular content beyond that envisaged necessary to attain competence according to the current CCF, underpinning a forward-thinking and globally relevant curriculum. Thorough, systematic quality assurance processes are integral to success of the new programme’s implementation.

## Appendix A

**Table A1:** Modules in each semester of the five-year programme.

**Year 1**	**Semester 1**	**Semester 2**
	General Principles of Pharmacology	Physiology
	From Molecules to Cells	Pharmaceutical Analysis 1
	Biochemistry	Introduction to Pharmaceutics and Formulation, including Mathematical Methods and Pharmaceutical Calculations
	Physical Pharmacy 1	Practice of Pharmacy 1
	Introduction to Pharmaceutics and Formulation, including Mathematical Methods and Pharmaceutical Calculations	Organic and Inorganic Chemistry for Pharmacy
	Practice of Pharmacy 1	
	Organic and Inorganic Chemistry for Pharmacy	
**Year 2**	**Semester 1**	**Semester 2**
	Blood, Cardiovascular and Renal Pharmacology and Clinical Therapeutics	Physical Pharmacy II, Drug Transport and Kinetics
	Properties and Analysis of Materials Used in Medicines	Properties and Analysis of Materials Used in Medicines
	Formulation and Pharmaceutical Technology	Formulation and Pharmaceutical Technology
	Practice of Pharmacy II	Practice of Pharmacy II
	Pharmaceutical Biochemistry and Biotechnology	Pharmaceutical Biochemistry and Biotechnology
	Molecular and Chemotherapeutic Pharmacology and Clinical Therapeutics	Molecular and Chemotherapeutic Pharmacology and Clinical Therapeutics
**Year 3**	**Semester 1**	**Semester 2**
	Neuropharmacology and Clinical Therapeutics	Natural Sources of Drugs and Substances used in Medicines
	Respiratory & Gastrointestinal Systems and Clinical Therapeutics	Endocrine & Reproductive Pharmacology and Clinical Therapeutics
	Sterile Products and Advanced Pharmaceutical Biotechnology	Malignant Disease, Immune & Ocular Systems and Clinical Therapeutics
	Practice of Pharmacy III	Sterile Products and Advanced Pharmaceutical Biotechnology
		Practice of Pharmacy III
**Year 4**	**Semester 1**	**Semester 2**
	Organisation and Management Skills	Advanced Pharmaceutical Chemistry, Drug Discovery & Design
	Personal Skills Development	Science Informing Practice
	Professional Practice	Research Project
**Year 5**	**Semester 1**	**Semester 2**
	Industrial Pharmacy	Supply of Medicines and Organisation and Management Skills
	Complementary and Alternative Medicine: Context, Legislation, Standards and Practice	Leading the Safe and Responsible Use of Medicines
	Advanced Pharmaceutics	Professional Practice and Public Health
	Practice of Pharmacy & Integrated Pharmacy Skills	Practice Research Project
	Addiction Pharmacy	Professional Registration Examination
